# Nutritional Management in a 101-Year-Old Woman with Physical Inactivity and General Weakness: A Case Report

**DOI:** 10.3390/geriatrics8010008

**Published:** 2023-01-06

**Authors:** Ryoko Mineyama, Fumie Tezuka, Nobuko Takagi, Shoichiro Kokabu, Masahiko Okubo

**Affiliations:** 1 Takagi Dental Clinic, Tsuchiura 300-0814, Japan; 2 Food and Nutrition Department, Koibuchi College of Agriculture and Nutrition, Mito 319-0323, Japan; 3 Division of Molecular Signaling and Biochemistry, Kyushu Dental University, Kitakyushu 803-8580, Japan; 4 Medical Corporation Association RISEIKAI, Tokyo 470-1141, Japan

**Keywords:** centenarian, managerial dietician, nutritional management, modified water swallowing test

## Abstract

Japan has the world’s highest life longevity, and centenarian patients are no longer rare. However, sufficient information related to centenarians is not available. Herein, we report the case of a 101-year-old centenarian woman who recovered from extreme inactivity and general weakness, mainly through nutritional management at home, to understand instances of nutritional management in centenarians. The patient developed lethargy, with a rapid decline in activity levels and food intake. She was diagnosed with senility by a primary doctor. We concluded that she had no problems with feeding and swallowing and predicted that her motivation to eat had decreased. We planned an intervention that lasted three months. To reduce the risk of aspiration, we paid attention to her posture while eating. To stimulate her appetite, we increased the variety and color of food items. To consider both the texture of food and safety, we changed the form of foods from paste (IDDSI Level 4)-like to solid food of regular size as much as possible. We recommended that the patient consume her favorite sweet between meals to enjoy eating. Two and half months after the initial intervention, the patient’s inactivity and general weakness improved dramatically, which was recognized by her willingness to eat, laugh loudly, and hum, although she could not speak clearly. The patient finally was able to have dinner with her family.

## 1. Introduction

The percentage of aging population has increased worldwide [1], and efforts by researchers and clinicians to study older adults [2,3,4] and centenarians [5,6,7] are increasing. Japan has the world’s highest life longevity, and centenarian patients are no longer rare. Tai et al. 2021 [8] showed that centenarians with lower calf circumference were associated with an increased risk of cognitive impairment, and underweight women were predisposed to cognitive impairment [5]. However, there is wide variation in the currently available data [9,10]. Management of centenarians and older adults differs considerably according to settings (e.g., hospital vs. residential), and strategies to improve muscle mass, muscle function, and quality of life (QOL) in this population have been investigated [11]. Malnutrition, especially in older adults, is recognized as a health problem associated with increased mortality and morbidity, as well as with physical decline affecting activities of daily living and general QOL [12]. In addition, the Japanese public nursing care insurance system was revised in 2021. Until now, home nutritional guidance by dietitians was not widely available because a visiting dietitian had to be affiliated with the same organization as the primary physician. The revised rule allows dietitians belonging to a different organization than that of the primary doctor to provide nutritional guidance [13]. Therefore, it is anticipated that home nutritional guidance will be widely adopted. However, the nutritional management system at home and the system of evaluating feeding and swallowing functions to support adequate food intake at home are insufficient. To understand the nutritional management of centenarians, we report the case of a 101-year-old centenarian woman who recovered at home from extreme inactivity and general weakness, mainly through nutritional management.

## 2. Case Report

A 101-year-old woman presented with a severe reduction in food intake. She had a history of ischemic stroke with left hemisphere paralysis and difficulty in speaking. One month before the initial examination, the patient developed lethargy, with a rapid decline in activity level and food intake. The primary doctor had diagnosed this condition as senility, and no further interventions were conducted to improve her condition. Therefore, the patient, with her family, consulted our team for further treatment. Our team, consisting of a dietitian, a dental hygienist, and a dentist, provides nutrition, feeding, and swallowing support for home care patients.

**Figure 1:**
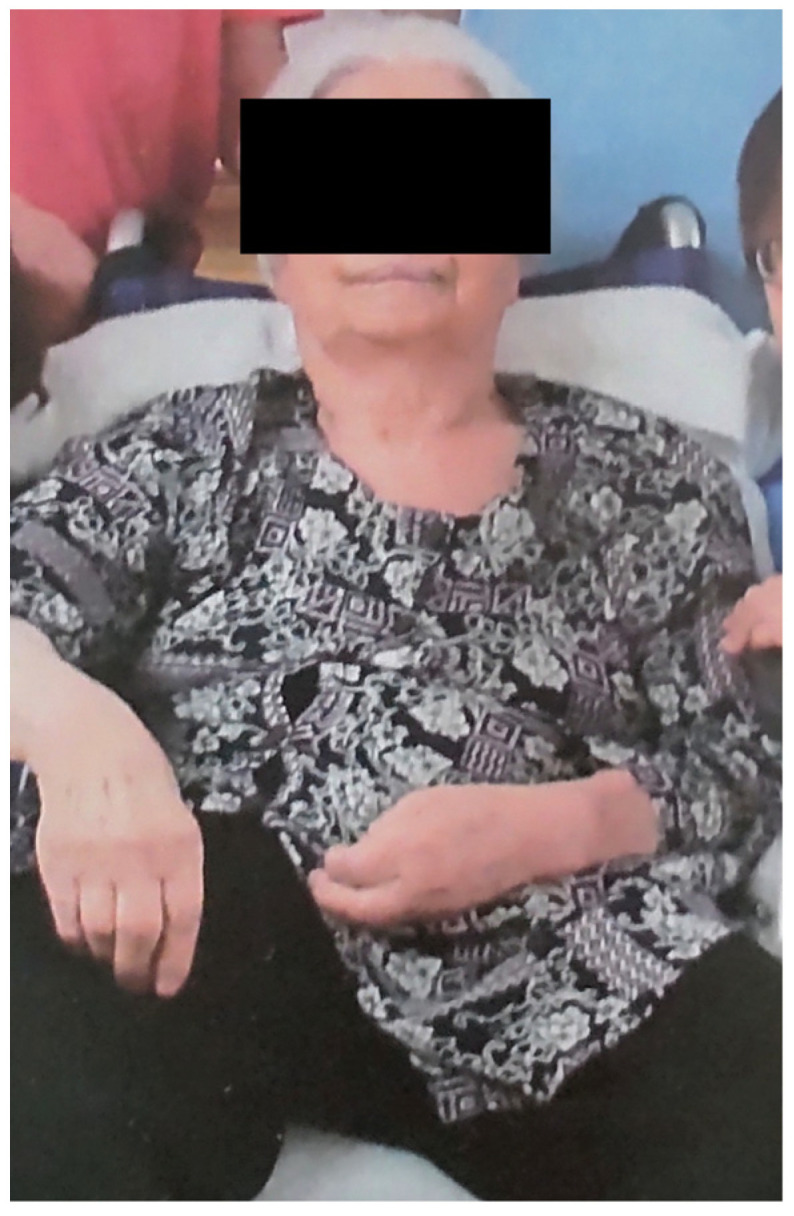
Frontal photograph of the patient during the initial examination.

**Figure 2:**
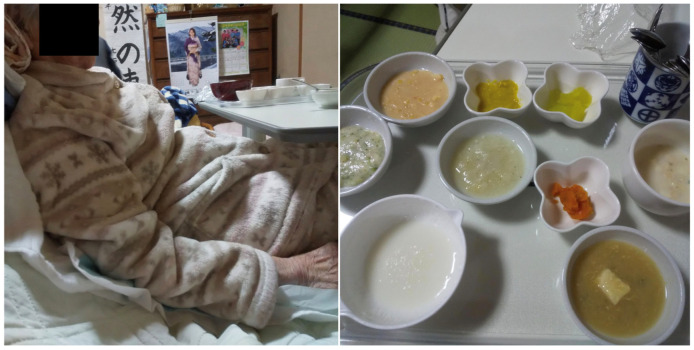
Positioning while eating (**left**) and meals (IDDSI Level 4) (**right**) at the first examination.

Initial investigations revealed a Glasgow Coma Scale score of E4V2M5, mild dementia, a height of 135 cm, and a body weight and body mass index (BMI) of 39.0 kg and 21.4 kg/m^2^, respectively; Her BMI seemed to be within normal; however, it should be interpreted with caution as there is much debate regarding the validity of BMI for adults over 65 years [14]. The patient had left hemiplegia (Brunnstrom stage: upper limb III, finger IV, and lower limb III), and the Barthel Index [15] was 5 points, indicating that she required full assistance with activities of daily living, including eating, going to the toilet, and bathing. The patient was taken care of by her son, daughter-in-law, and grandchildren. The patient had difficulty standing; however, she could sit (Figure 1). Although blood examination revealed slight abnormalities in several parameters, such as the number of red blood cells, total protein, albumin, blood urea nitrogen (BUN), blood glucose, triglycerides, total cholesterol, and low-density lipoprotein cholesterol, she had no apparent systemic diseases (Table 1) or motor impairment of the facial muscles. Further, no apparent decline in tongue function and no obvious curtain signs on the posterior wall of the pharynx were noted. The modified water swallowing test score was 5 [16]. Cervical auscultation revealed no prominent murmur when the patient drank water. These findings indicated that she did not have dysphagia. The oral environment was relatively good, as there was no evident dryness or contamination. Although she was edentulous, there was no problem with the functioning of her denture, indicating that she had no abnormalities in her eating function. However, her eating posture was evaluated as poor, based on the following findings: her pelvic girdle was tilted backwards, resulting in a backward center of gravity, and the upper chest, head, and neck were deviated forward to maintain total postural balance. We predicted that while eating in such a posture, the mandible protruded forward, and the mobility of the pharynx and larynx reduced because of the neck extension. Furthermore, we also predicted that the mandible protrusion stretched the sublingual muscles and restricted the function of the supraglottic muscles to elevate the larynx (Figure 2 left). All her food items were paste (International dysphagia standardization Initiative; IDDSI Level 4)-like, colorless, and unappetizing (Figure 2 right). Hence, we concluded that she had basically no problems with feeding and swallowing, and predicted that her motivation to eat was declining.

We started the intervention by visiting the patient’s home once per week. She did not have a significant loss of body weight. There was no hypertension and there were no renal problems; therefore, we did not restrict the salt or protein content in the patient’s diet. We targeted a total energy intake of 1000 kcal/day based on the Harris and Benedict equations as a reference, although this equation applies to people aged 21–70 years with a weight of 25.0–124.9 kg and a height of 151–200 cm, and was not applicable for our patient [17]. Furthermore, much effort was devoted to menu planning to stimulate appetite safely.

**Figure 3:**
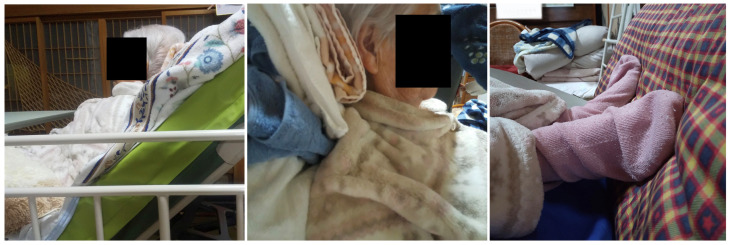
Side view (**left**), neck region (**middle**), and foot region (**right**) of the patient after postural regulation.

To reduce the risk of aspiration and avoid compression of the lumbar spine, her bed was set at a 40° recline angle (Figure 3 left). The knees were bent lightly by placing cushions beneath the bottom of her feet (Figure 3 middle), and her head was stabilized, with the chin slightly pulled back using a bath towel (Figure 3 right).

To support her eating, we used a spoon that stimulated her K-points. The K-point lies on the mucosa lateral to the palatoglossal arch and medial to the pterygomandibular fold at the height of the post-retromolar pad. When this point is stimulated, jaw opening, and swallowing are induced [18]. We attempted to prevent aspiration, due to cervical extension, by carrying food from a position below the patient’s visual line. We inserted a spoon with food into her mouth, parallel to the dorsum of her tongue. Before pulling out the spoon, we induced pressure stimulation and lip closure by contacting the bottom of the spoon with the center of the tongue dorsum.

To stimulate her appetite, we increased the variety and color of her food items. To consider both texture of food and safety, we changed the form of foods from paste (IDDSI Level 4)-like to solid food of regular size as much as possible. We took advantage of the commercially available high-calorie jelly food ENEPURIN^®^ (The Nisshin OilliO Group, Ltd., Tokyo, Japan) to adjust calorie intake. The protein requirement was 39 g, and this was calculated by regarding the current body weight as the target. Because the diet alone was 10 g deficient in the target value, this deficiency was supplemented with whey protein in each meal. Furthermore, we recommended that she eat her favorite sweets between meals to enjoy eating. 

**Figure 4:**
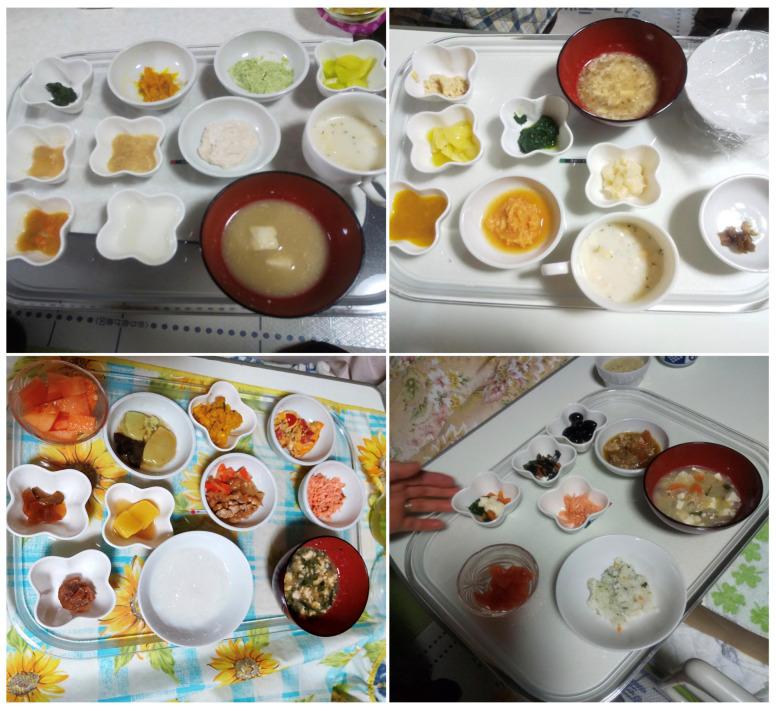
Changes in meals at 2 weeks (IDDSI Level 4) (**upper left**), 1 month (IDDSI Level 5) (**upper right**), 1.5 months (IDDSI Level 6) (**lower left**), and 2.5 months (IDDSI Level 7) (**lower right**) after the initiation of the intervention.

The transition in meals after the initiation of the intervention is presented in Figure 4. Immediately after the initial intervention, the food leaked out of the patients’ oral cavity because the patient kept sticking her tongue out, and only a mouthful of food was ingested. One week after the initial intervention, five or six mouthfuls of food were ingested by the patient at one mealtime. She was able to finish a Japanese cake.

Two and half months after the initial intervention, the patient could finish her meals and achieve the calorie intake goals. The patient’s extreme inactivity and general weakness improved dramatically, which was recognized by her willingness to eat, her loud laugh, and her humming, even though she could not speak clearly. The patient was able to have dinner with her family. To date, no aspiration or other problems have occurred.

## 3. Discussion

In this case, we achieved a remarkable recovery in a 101-year-old woman with extreme inactivity and general weakness by home care and support centered on nutritional guidance. Therefore, our case may contribute to establishing a system for at-home nutritional management of centenarians.

The advantages of home care include the ability to stay in a familiar environment and provide food with a familiar taste. However, unlike the situation in hospitals, patients must spend most of their time without a dietician or medical professional at home. Therefore, cooperation among those living with the patients is essential for food support. The study by Barrado-Martín et al. in 2022 [19] indicated the importance of professionals providing education and support to family caregivers for the nutrition of people with dementia. Therefore, the role of visiting staff, including visiting managerial dieticians, dental hygienists, and dentists, may be crucial in educating people about food support. Whenever we visited, we not only examined the patient’s condition, but also consulted with the caregivers and provided them with appropriate instructions and information on food support.

There is a well-established limitation on the quantity and quality of per-meal and total food intake for older adults [20], and the consensus on the importance of adjusting the diet, especially the supply of protein, for muscle health [21]. However, recommending protein supplementation as a stand-alone intervention for healthy older individuals seems ineffective in improving muscle mass and strength [22]. Therefore, the ideal strategy is the combination of exercise and adequate protein intake to mitigate the damage of aging in multiple contexts [23,24].

Eating well brings spiritual richness and satisfaction and helps establish social relationships and communication. Eating is considered a major source of recreation in older adults, including centenarians, because their social activity is usually restricted. In 2004, Yoshihara A. et al. [25] reported a positive correlation between appetite and QOL in a study of a Japanese older adults’ community [25]. If we are obliged to eat even if we did not enjoy the food, simply to sustain life, then the act becomes painful. The meal must taste and look satisfactory. Older adults who have difficulty chewing and swallowing are often offered paste (IDDSI Level 4) meals, mixer meals (IDDSI Level 4), or other foods that are not in their original form.

The pattern of eating in older adult individuals is different from that of infants at the beginning stages of eating. Older adults have had good food in the past. The purpose of a meal is not merely to meet the nutrient and energy requirements. The European Pressure Ulcer Advisory Panel’s guidelines for pressure ulcer prevention and treatment include considerations for appetizing food with attractive presentations [26]. Recently, considering this, appetizing soft food is being provided. These foods are commercially available. Studies have shown that ordinary soft foods are more satisfying than paste (IDDSI Level 4) meals [27]. Therefore, we actively offered the patient’s favorite food. Attention was also paid to meal color. The foods were made similar to regular meals by making them soft and seasoning them with familiar flavors.

In addition to focusing on the texture and form of meals, other methods can be used to prevent aspiration and support eating. To prevent aspiration and aspiration pneumonia, proper positioning of the neck, trunk, and extremities is considered effective [28].

## 4. Conclusions

A multidisciplinary intervention combined with adequate nutritional management was able to mitigate a scenario of extreme inactivity and general weakness. In this study, we reiterated the importance of focusing on QOL and ensuring sufficient nutrition to support the physical and mental needs of older adults, especially centenarians.

## Figures and Tables

**Table 1:** Blood test results at the initial examination.

Item	Value	Normal Range	Item	Value	Normal Range
White blood cell	64	35~91 (10^2^/mL)	Creatinine	0.64	0.45~0.82 (mg/dL)
Red blood cell	358	376~500 (10^4^/mL)	Blood urea nitrogen (BUN)	24.4	8.0~23.0 (mg/dL)
Hemoglobin	11.8	11.3~15.2 (g/dL)	Blood glucose	130	70~109 (mg/dL)
Hematocrit	37.2	33.4~44.9 (%)	Hemoglobin A1c	5.3	4.6~6.2 (%)
Platelet	16.3	13.0~36.9 (10^4^/mL)	TG	214	35~149 (mg/dL)
Total protein	6.1	6.7~8.3 (g/dL)	Total cholesterol	227	120~219 (mg/dL)
Albumin	3.6	3.8~5.3 (g/dL)	LDL cholesterol	143	70~139 (mg/dL)
Aspartate aminotransferase (AST)	30	10~40 (U/L)	Natrium	143	134~147 (mEq/L)
Alanine aminotransferase (ALT)	24	5~45 (U/L)	Potassium	3.8	3.4~5.0 (mEq/L)
Lactase dehydrogenase (LDH)	154	120~240 (U/L)	Chloride	105	98~108 (mEq/L)
Alkaline phosphatase gene (ALP)	325	104~338 (U/L)	Total bilirubin	0.39	0.20~1.10 (mg/dL)
γ-glutamyl transpeptidase (γ-GTP)	16	0~35 (U/L)			

## Data Availability

Not applicable.
